# Estimation of influenza and respiratory syncytial virus hospitalizations using sentinel surveillance data—La Paz, Bolivia. 2012–2017

**DOI:** 10.1111/irv.12663

**Published:** 2019-06-17

**Authors:** Dabeyva Chavez, Vicente Gonzales‐Armayo, Elvis Mendoza, Rakhee Palekar, Rosario Rivera, Angel Rodriguez, Claudia Salazar, Angel Veizaga, Arletta Añez

**Affiliations:** ^1^ Minister of Health La Paz Bolivia; ^2^ Servicio Departamental de Salud La Paz Bolivia; ^3^ Pan American Health Organization WDC USA; ^4^ Instituto de Laboratorios en Salud INLASA La Paz Bolivia; ^5^ Hospital Boliviano Holandés El Alto Bolivia; ^6^ Pan American Health Organization La Paz Bolivia

**Keywords:** Bolivia, influenza, respiratory syncytial virus, SARI

## Abstract

**Objective:**

The objective was to estimate the number of hospitalizations associated with influenza and RSV using data from severe acute respiratory infection (SARI) sentinel surveillance from El Alto‐La Paz. Bolivia.

**Methods:**

All persons who met the case definition for SARI at one sentinel hospital had a clinical sample collected and analyzed by rRT‐PCR for influenza and by indirect immunofluorescence for RSV. The SARI‐influenza and SARI‐RSV case counts were stratified by six age groups. The proportion of cases captured in the sentinel hospital in relation to the non‐sentinel hospitals of area was multiplied by the age‐specific census population, to build the denominators. The annual incidence and a 95% confidence interval (CI) were estimated.

**Results:**

During 2012‐2017, n = 2606 SARI cases were reported (average incidence 120/100 000 inhabitants [95% CI: 116‐124]); the average incidence of influenza‐associated SARI hospitalization was 15.3/100 000 (95% CI: 14.1‐16.7), and the average incidence of RSV‐associated SARI hospitalization was 9/100 000 inhabitants (95% CI: 8.1‐10.1). The highest incidence of influenza was among those less than one year of age (average 174.7/100 000 [range: 89.1‐299.5]), followed by those one to four years of age (average 51.8/100 000 [range: 19.8‐115.4]) and then those 65 years of age and older (average 47.7/100 000 [range: 18.8‐117]). For RSV, the highest incidence was highest among those less than one year of age (231/100 000 [range: 119.9‐322.9]).

**Conclusion:**

Influenza and RSV represent major causes of hospitalization in La Paz, Bolivia—with the highest burden among children under one year of age. Our estimates support current prevention strategies in this age group.

## 1 BACKGROUND

Acute respiratory infections (ARI) are one of the main causes of morbidity and mortality worldwide. ARIs affect preferentially and with greater severity, vulnerable populations such as pregnant women, chronically ill people, older adults, and children under 5 years of age, especially in lower‐middle and low‐income countries.^1^, ^2^, ^3^, ^4^ Influenza and respiratory syncytial virus (RSV) are two of the main etiological agents causing ARIs in all age groups and primarily among children, respectively. Both of these viruses are associated with high transmissibility and virulence. The influenza virus also has the ability to mutate and cause pandemics and challenges associated with immunity and vaccine response.^5^ Knowing the burden of disease associated with these viruses is relevant for countries, since it allows implementation of preventive and control measures and the development of vaccine cost‐effectiveness and economic burden studies among others.^6^, ^7^, ^8^

Despite the importance of disease‐burden estimation, the quantification is complex due to the low specificity of clinical symptoms, the broad spectrum of clinical illness, the presence of co‐infections, the lack of routine laboratory analysis of patients with acute respiratory symptoms, and the poor quality of clinical records.^7^, ^8^, ^9^ In order to measure the incidence of influenza and other respiratory viruses, different methodologies have been utilized. Some of these methodologies use data from sentinel influenza surveillance for severe acute respiratory infections (SARI) or influenza‐like illness (ILI), and others use hospital admissions or discharges paired with population demographics.^9^, ^10^ Historically in the Americas, most respiratory virus burden estimation studies were conducted in the United States of America 11, 12, 13, 14; however, since the 2009 influenza pandemic, in Latin America, sentinel surveillance of SARI has improved, which has resulted in higher quality surveillance data and increased identification of etiologic agents and together, which has made it possible to estimate the burden of disease associated with influenza and RSV. 15, 16, 17, 18, 19

In Bolivia, in 2011 SARI sentinel surveillance was implemented among eight sentinel hospitals—five in the city of La Paz and three in the city of Santa Cruz, to monitor influenza and RSV circulation seasonality disease severity and patient risk factors. In this analysis, we sought to estimate the burden of hospitalizations associated with influenza and RSV in El Alto‐La Paz, Bolivia, using SARI sentinel surveillance data from the Hospital Boliviano Holandés (HBH) during 2012‐2017.

## 2 METHODS

The municipality of El Alto is located within the metropolitan area of the city of La Paz, which is located in the Andean region of Bolivia at an altitude of 4150 m. This region covers 28% of the area of the country and is inhabited by approximately 39.7% of the Bolivian population (11 million persons). El Alto has five hospitals that treat patients with ARIs including one SARI sentinel hospital, HBH.

SARI surveillance is conducted using the WHO SARI case definition—history of fever or measured fever of ≥38°C, cough, hospitalization, and onset of symptoms in the last ten days.^20^ Clinical samples are collected from all persons that meet the SARI case definition, and in the case of HBH, clinical samples are processed in the national laboratory INLASA, which has diagnostic capacity for indirect immunofluorescence for RSV and real‐time reverse transcriptase‐polymerase chain reaction (rRT‐PCR) for influenza.

### 2.1 Calculating the number of influenza and RSV cases

The total number of SARI cases positive for influenza and RSV was counted for each year of the analysis. Only cases with residence in El Alto were included in the case count.

### 2.2 Estimating the proportion of the catchment area covered by HBH

The catchment area of HBH was considered to be the municipality of El Alto, whose 2012 population was available from the National Statistics Institute (INE). In total, there were four non‐sentinel and one sentinel hospitals that admit persons with respiratory disease in this catchment area. In order to ascertain the proportion of the catchment area covered by HBH, we calculated the annual number of SARI hospitalizations by age at HBH relative to these five hospitals. In order to calculate the number of respiratory hospitalizations at the non‐sentinel sites hospitals in the catchment area, we used SARI‐proxy International Classification of Diseases 10th edition (ICD‐10) codes J09‐22 by age.^9^ Then, for each year of the analysis, we divided the number of SARI hospitalizations at HBH by the sum of the SARI‐proxy codes at all five hospitals as well as the SARI cases from HBH. We applied the resulting proportion to the 2012‐census population of El Alto, in order to adjust the census population to reflect the catchment area population of HBH.^9^

### 2.3 Age groups

We analyzed all data using the following six age groups: under one year of age, one through 4 years of age, five through 19 years of age, 20 through 49 years of age, 50 through 64 years of age, and 65 + years.

### 2.4 Estimating the incidence

Hospitalization incidence was estimated for influenza‐ and RSV‐associated SARI for each year and age group per 100 000 inhabitants by dividing the number of influenza or RSV SARI cases at HBH by the adjusted census population. A 95% confidence interval (95% CI) was calculated around the point estimate.

### 2.5 Statistical analysis

Excel and Tableau® 10 (Professional Edition) were used for descriptive analyses.

## 3 RESULTS

### 3.1 Distribution of SARI cases

During 2012 through 2017, n = 2784 SARI cases were reported from HBH. We excluded n = 178 of these cases as they were not residents of El Alto, leaving 2606 valid cases in the study. In total, 99.5% (n = 2592) of the SARI cases had clinical samples collected, 98.9% (n = 2565) were tested by rRT‐PCR and 97.3% (n = 2523) were tested by IFA.

**Figure 1:**
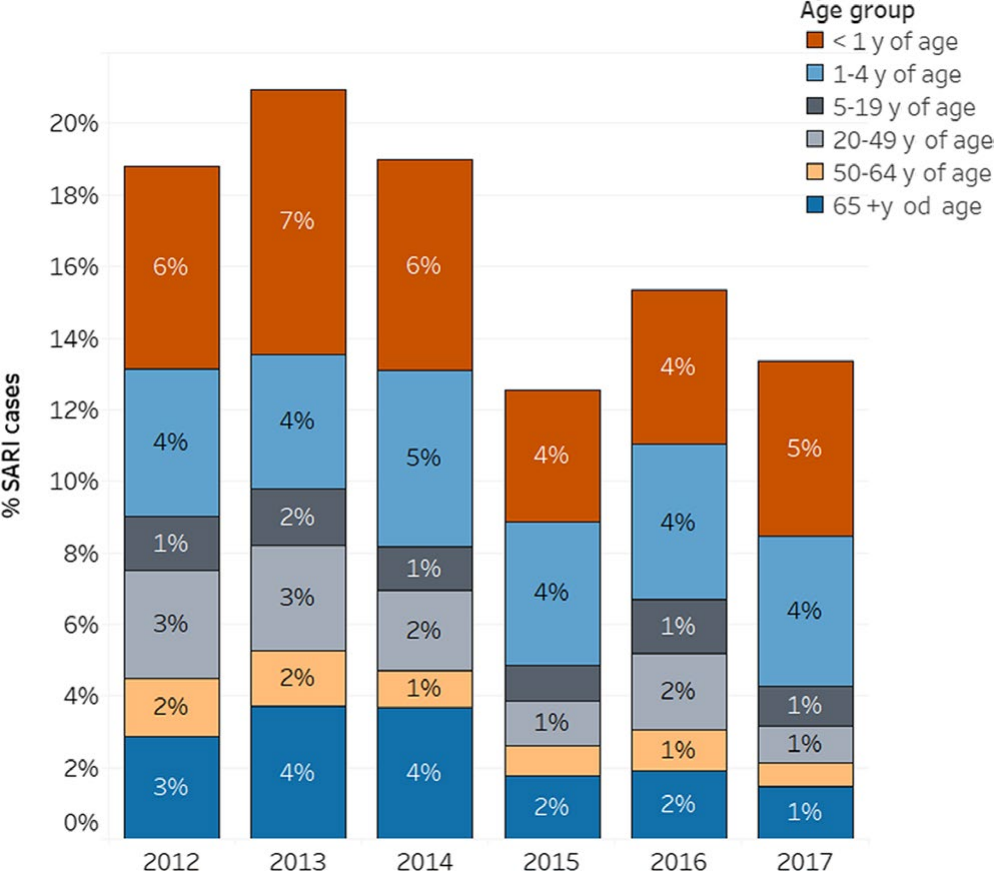
SARI cases by age group—Hospital Boliviano Holandés. Bolivia. 2012‐2017

Among the SARI cases included in the analysis (n = 2592), 31.9% were younger than one year of age, 25.3% were between 1 and 4 years of age, 7.9% were between 5 and 19 years of age, 12.6% were between 20 and 49 years of age, 7.9% were between 50 and 64 years of age, and 15.4% were 65 years of age or older. The highest number of SARI cases was reported in 2013 (n = 545), while the lowest number was reported in 2015 (n = 327) (Figure 1).

### 3.2 Viral circulation

The overall positivity for influenza was 12.5% (327/2606), with a minimum of 8.3% (27/327) in 2015 and a maximum of 21.8% (87/400) in 2016, while for RSV, the overall positivity was 7.9% (205/2606) with a minimum of 4.0% (16/400) in 2016 and a maximum of 13.5%(67/495) in 2014.

**Figure 2:**
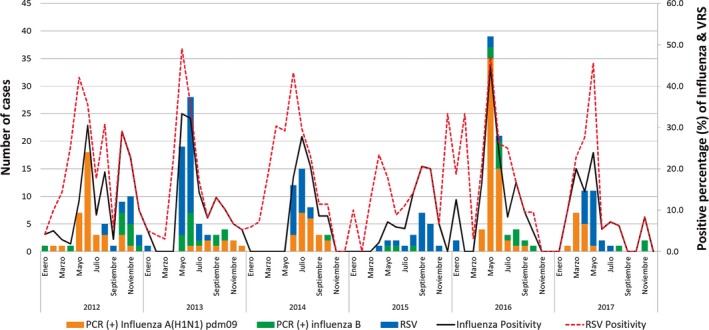
Distribution of RSV and influenza cases by type and subtype—Hospital Boliviano Holandés. Bolivia. 2012‐2017

Among all influenza cases, the majority of cases were influenza A(H1N1)pdm09 45.6% (154/338), followed by influenza A (H3N2) 35.8% (121/338) and finally influenza B 15.4% (52/338); 3.3% (11/338) of cases were influenza A that were not subtyped. Influenza A (H1N1)pdm09 predominated in 2012, 2014, and 2016 (53.5%. 53.5%, and 79.3% of 154 samples, respectively); influenza A (H3N2) predominated in 2013, 2015, and 2017 (58.1%. 74.1%, and 52.8% of 121 samples, respectively); and influenza B did not predominate during any of the years of the analysis (Figure 2).

### 3.3 SARI, influenza, and RSV hospitalization incidence

The average SARI incidence during 2012‐2017 was 120/100 000 inhabitants (95% CI: 117‐124). The average influenza‐associated hospitalization incidence during 2012‐2017 was 15.3 per 100 000 inhabitants (95% CI: 14.1‐16.7) and for RSV was nine per 100 000 inhabitants (95% CI: 8.1‐10.1) (Table 1).

**Table 1:** Incidence of SARI. Influenza and RSV hospitalizations per thousand inhabitants—Hospital Boliviano Holandés. Bolivia. 2012‐2017

Age group	SARI incidence (95% CI)†	Influenza incidence (95% CI)†	RSV incidence (95% CI)†
2012			
<1 y	1567.6 (1352.8‐1816.4)	168.3 (107.3‐263.8)	283.4 (200.4‐400.8)
1‐4 y	215.4 (177.4‐261.5)	31.7 (19.1‐52.5)	23.2 (12.9‐41.9)
5‐19 y old	19.2 (13.9‐26.5)	3.1 (1.4‐6.9)	1 (0.3‐4.1)
20‐49 y old	28.7 (22.2‐37.2)	5 (2.7‐9.2)	0
50‐64 y old	28.7 (22.2‐37.2)	5 (2.7‐9.2)	0
≥65 y	474 (378‐594.4)	63.2 (34‐117.5)	0
2013			
< 1 y	1772.1 (1556.8‐2017.1)	170.2 (112.1‐258.5)	193.5 (130.7‐286.3)
1 to 4 y	227.1 (185.5‐278)	41.1 (25.5‐66.1)	9.7 (3.6‐25.8)
5 to 19 y old	27.8 (20.2‐38.2)	3.7 (1.5‐8.8)	0
20 to 49 y old	37.6 (28.2‐50)	5.6 (2.7‐11.7)	0.8 (0.1‐5.7)
50‐64 y old	37.6 (28.2‐50)	5.6 (2.7‐11.7)	0.8 (0.1‐5.7)
≥65 y	756.2 (619.8‐922.7)	116.9 (70.5‐194)	7.8 (1.1‐55.3)
2014			
<1 y	1859.2 (1597.6‐2163.7)	89.1 (44.5‐178.1)	322.9 (224.4‐464.6)
1‐4 y	345.7 (290.7‐411.1)	40.5 (24.4‐67.2)	86.4 (61.1‐122.2)
5‐19 y old	22.1 (15.1‐32.5)	4.3 (1.8‐10.2)	0.9 (0.1‐6)
20‐49 y old	22.3 (16.9‐29.3)	3.9 (2‐7.6)	1.7 (0.7‐4.7)
50‐64 y old	22.3 (16.9‐29.3)	3.9 (2‐7.6)	1.7 (0.7‐4.7)
≥ 65 y	582.2 (476.7‐711.1)	24.3 (9.1‐64.6)	6.1 (0.9‐43.1)
2015			
<1 y	1590.3 (1309.8‐1931)	109.1 (52‐228.9)	171.5 (95‐309.7)
1‐4 y	340.2 (280.5‐412.7)	19.8 (8.9‐44.1)	33 (17.8‐61.4)
5‐19 y old	22.2 (14.9‐33.1)	0.9 (0.1‐6.6)	0.9 (0.1‐6.6)
20‐49 y old	19.7 (13.7‐28.1)	3.9 (1.8‐8.8)	0
50‐64 y old	19.7 (13.7‐28.1)	3.9 (1.8‐8.8)	0
≥65 y	478.2 (358.2‐638.4)	20.8 (5.2‐83.1)	10.4 (1.5‐73.8)
2016			
<1 y	1766.8 (1475.1‐2116.2)	299.5 (193.2‐464.2)	119.8 (59.9‐239.5)
1‐4 y	506.1 (421.2‐608)	115.4 (78.6‐169.5)	26.6 (12‐59.3)
5‐19 y old	34.8 (25.1‐48.3)	11.6 (6.6‐20.4)	1 (0.1‐6.9)
20‐49 y old	31.6 (24.1‐41.5)	8.5 (5‐14.4)	0.6 (0.1‐4.3)
50‐64 y old	31.6 (24.1‐41.5)	8.5 (5‐14.4)	0.6 (0.1‐4.3)
≥65 y	449.4 (340.6‐592.9)	35.9 (13.5‐95.8)	0
2017			
<1 y	2148.3 (1810.2‐2549.6)	196.8 (111.8‐346.5)	295.2 (186‐468.5)
1‐4 y	437.2 (362.4‐527.5)	51.2 (29.1‐90.2)	11.8 (3.8‐36.6)
5‐19 y old	25.9 (17.6‐38)	5 (2.1‐12)	0
20‐49 y old	11.9 (8.1‐17.5)	1.4 (0.4‐4.3)	0
50‐64 y old	11.9 (8.1‐17.5)	1.4 (0.4‐4.3)	0
≥65 y	178.9 (130.2‐245.8)	18.8 (7.1‐50.2)	0
Average rate 2012‐2107	120.2 (116.6‐124)	15.3 (14.1‐16.7)	9 (8.1‐10.1)

**Figure 3:**
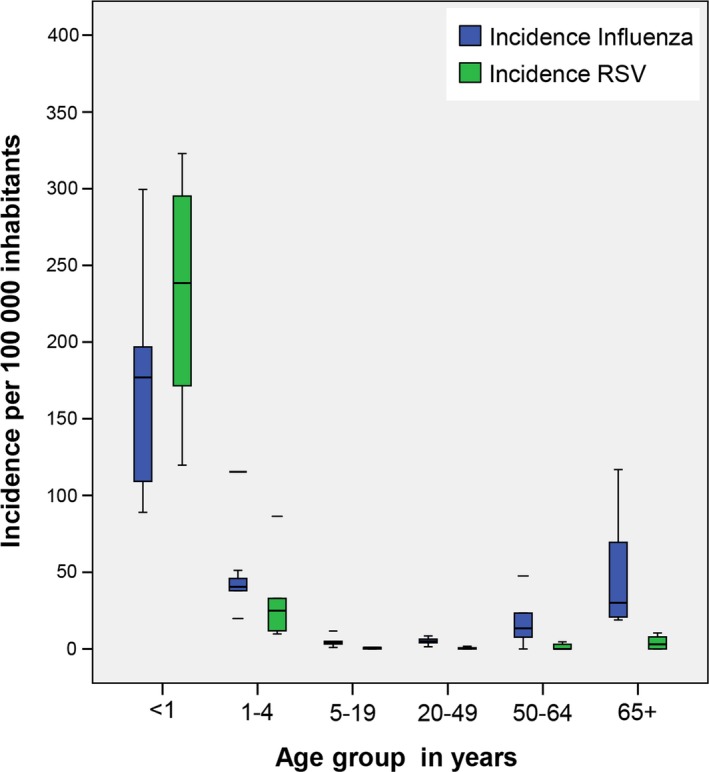
Incidence of influenza‐ and RSV‐associated SARI by age group—Hospital Boliviano Holandés. Bolivia. 2012‐2017

The highest incidence of influenza was among those under 1 year of age (average 174.7/100 000 inhabitants [range: 89.1‐299.5]), followed by those one to 4 years of age (average 51.8/100 000 inhabitants [range: 19.8‐115.4]) and followed by those 65 years of age and older (average 47.7 [range: 18.8 ‐ 117]) (Figure 3).

For RSV, the highest incidence was also among those less than 1 year of age (average 231/100 000 [range: 119.9‐322.9]), followed by those 1‐4 years of age (average: 31.8/100 000 [range: 9.7‐86.4]) (Figure 3).

## 4 DISCUSSION

In this analysis, we demonstrate the important hospitalization burden associated with influenza and RSV in La Paz, Bolivia. There are three important findings from this analysis that include the disproportionate influenza and RSV hospitalization burden seen among young children, the alignment of these estimates with those from other countries in this region, and the support that these estimates for influenza provide for current seasonal influenza vaccination strategies.

First, we observed the highest incidence of influenza‐associated hospitalizations among infants, over the course of the time series. The overall magnitude of the burden was similar from year to year, regardless of the influenza virus that predominated, with the exception of 2016, when the incidence was the highest. This finding of greatest burden in infants has been seen in other analyses, using alternate methodologies, in other countries with comparable low‐middle income status 17, 18, 21 and provides further evidence to support the current policy in Bolivia of targeted vaccination of this risk group with the seasonal influenza vaccine annually.

Next, the incidence of influenza‐associated hospitalizations among non‐infant age groups was comparable to estimates generated in other analyses in other low‐middle income countries.^17^, ^18^, ^21^ Specifically, of note, the incidence among the elderly was lower than what has been documented in higher‐income countries,^22^, ^23^ which could reflect a lower burden of hospitalizations due to better seasonal influenza vaccine coverage; lower risk‐factor burden and hence lower risk of severe outcome; earlier treatment in illness course with oseltamivir; demographic structure, inclined to young people;^28^ or might reflect different health‐seeking behavior and hospitalization threshold. Indeed, the alternate health‐seeking behavior and hospitalization threshold hypothesis has been substantiated in other analyses 24, 25, 26, 27 and is further supported by the overall higher influenza‐associated mortality incidence in this age group as compared to other age groups, as has been documented in other analyses.^16^, ^19^, ^23^

Finally, with regard to RSV hospitalizations, the clear burden was among infants, with zero cases reported among adults during several years of the time series. This burden among infants has been well documented in other analyses^3^, ^29^, ^30^; however, none of these analyses have taken place in low‐middle–income countries in Latin America. This burden of RSV among infants further emphasizes the need to develop and license effective vaccines that target pregnant women and infants.

With regard to the methodology used to estimate the hospitalization burden, as mentioned previously, the estimation of influenza and RSV disease burden in low‐middle–income countries has been challenging, due to lack of resources and secondarily, limited surveillance, and limited cohort analyses. In the last five years, however, in the context of improving surveillance, Latin American countries in collaboration with partners, such as the Pan American Health Organization (PAHO) and the US Centers for Disease Control and Prevention(CDC), have conducted analyses using sentinel syndromic surveillance data and direct ascertainment methods, as was done in this analysis, as well as using ICD‐10 coded hospital discharge data paired virologic surveillance data, in an attribution‐of‐cases method. At this time, it is still uncertain what is the best method to measure influenza hospitalizations burden, as there are no published comparisons of these methods to date using data from Latin America.

This analysis is subject to at least four limitations. First, we assumed that all SARI‐influenza and SARI‐RSV cases were captured at the sentinel site. While thorough assessment is completed periodically by the national and local surveillance team to ensure SARI case capture, it is unlikely that all SARI cases were captured, and as such, our estimated incidence might be lower than the true incidence; along these same lines, the SARI case definition being used to identify possible influenza and RSV cases might not be equally sensitive for influenza and RSV among all age groups, further lowering our incidence estimate compared to the true burden. Next, as mentioned previously, it is likely that we underestimated the burden of influenza and RSV among the elderly due to specific health‐seeking behavior practices as well as local hospitalization thresholds. Finally, specifically with regard to RSV burden, as IFA, which has a lower sensitivity than rRT‐PCR, was used to confirm the virologic etiology of the cases,^3^, ^29^ it is likely that we underestimated the RSV burden. As a final note, although not the objective of this analysis and hence not a true limitation, with these data, we were unable to extrapolate to the national population of Bolivia, as HBH might not be representative of all of the other public and private hospitals in Bolivia.

In conclusion, this is the first analysis to document the hospitalization burden of influenza and RSV in La Paz. Bolivia. These data provide the much‐needed justification for ongoing investments in prevention and control strategies, such as seasonal influenza vaccine, and provide the data needed to complete influenza vaccine impact analyses and to generate influenza and RSV economic burden estimates. Future analyses should focus on generating national estimates of hospitalization burden in Bolivia, as well as estimating seasonal influenza vaccine impact and economic burden of influenza and RSV.

## CONFLICT OF INTERESTS

The authors declare that they have no conflict of interest.

## AUTHOR CONTRIBUTIONS

CD, EM, and AA conceptualized the study. AA wrote the draft manuscript and interpreted the data. P.R, RA, and GA critically reviewed the manuscript, and SC, QR,A and VA involved in the development of the plan of analysis, primary data collection and analysis, data interpretation, and critically reviewed the final report. All authors read and approved the final draft of the report.
